# Assessing developmental toxicity of caffeine and sweeteners in medaka (*Oryzias latipes*)

**DOI:** 10.1186/s40064-015-1284-0

**Published:** 2015-09-08

**Authors:** Wenjau Lee, Yun-Chi Wang

**Affiliations:** Department of Bioscience Technology, Chang Jung Christian University, No. 1, Changda Rd., Gueiren District, Tainan, Taiwan

**Keywords:** Sweetener, Caffeine, Medaka, Behavior, Developmental toxicity

## Abstract

The use of artificial sweeteners (ASWs) has increased and become more widespread, and consequently ASWs have appeared in aquatic environments around the world. However, their safety to the health of humans and wildlife remains inconclusive. In this study, using medaka embryos (*Oryzias latipes*), we investigated developmental toxicity of aspartame (ASP) and saccharin (SAC). Since ASWs are often consumed with caffeine (CAF) and CAF with sucrose (SUC), we tested biological activities of these four substances and the mixtures of CAF with each sweetener. The embryos were exposed to ASP at 0.2 and 1.0 mM, SAC at 0.005 and 0.050 mM, CAF at 0.05 and 0.5 mM, or SUC at 29 and 146 mM, starting from less than 5 h post fertilization until hatch. Control embryos were treated with embryo solution only. Several endpoints were used to evaluate embryonic development. Some of the hatchlings were also tested for anxiety-like behavior with the white preference test. The results showed that all four substances and the mixtures of CAF with the sweeteners affected development. The most sensitive endpoints were the heart rate, eye density, and hatchling body length. The hatchlings of several treatment groups also exhibited anxiety-like behavior. We then used the Integrated Biological Response (IBR) as an index to evaluate the overall developmental toxicity of the substances. We found that the ranking of developmental toxicity was SAC > CAF > ASP > SUC, and there was a cumulative effect when CAF was combined with the sweeteners.

**Electronic supplementary material:**

The online version of this article (doi:10.1186/s40064-015-1284-0) contains supplementary material, which is available to authorized users.

## Background

Artificial sweeteners (ASWs) have been in use for decades, but they are now increasingly added to all kinds of foods, drinks, and pharmaceutical products. As a consequence, ASWs are excreted from our bodies and discharged with sewage treatment effluents to the aquatic environment. Therefore, they have emerged as a class of environmental contaminants.

For example, saccharin (SAC) is largely unmetabolized in human body, allowing it to pass unchanged to the environment, mainly through the urine. It is usually degraded by more than 90 % during wastewater treatment (Lange et al. 2012). However, wastewater is not always properly treated, and SAC concentrations may be too high to be efficiently removed. In a Canadian river watershed, SAC at a concentration of 7.2 µg/L was found where both urban population and the consumption of calorie-reduced beverages were high (Spoelstra et al. 2013). Concentrations of SAC up to 19.7 µg/L were also found from surface waters in Spain (Ordóñez et al. 2012), and up to 137 µg/L from wastewater in Singapore (Tran et al. 2013).

On the other hand, aspartame (ASP) is metabolized to 50 % phenylalanine, 40 % aspartic acid, and 10 % methanol in humans (Ranney et al. 1975). The small amount of methanol has been suggested to be responsible for ASP carcinogenicity (Soffritti et al. 2010). Because these components are absorbed and metabolized as other foods, ASP is usually not detected in environmental water samples (Lange et al. 2012; Ordóñez et al. 2012). Nonetheless, Gan et al. (2013) have reported its presence in surface waters at concentrations up to 0.21 µg/L in China.

Unlike other contaminants, ASWs are substances we actively purchase and ingest regularly, sometimes in large quantities. Although many studies have supported the safety and benefits of ASWs (Weihrauch and Diehl 2004; Marinovich et al. 2013), clear evidence of safety in long-term use is still lacking (Wiebe et al. 2011; Shankar et al. 2013; Gardner 2014). In addition, their developmental toxicity is surprisingly inconclusive. For example, prospective studies on pregnant women have found an association of daily intake of ASWs-containing beverages with preterm delivery (Halldorsson et al. 2010; Englund-Ögge et al. 2012) or offspring allergic diseases (Maslova et al. 2013), but Marinovich et al. (2013) have also concluded in their review study that ASWs were not related to preterm delivery.

Animal studies on developmental toxicity of ASWs are mostly conducted on rats and mice; studies on fish or aquatic organisms are scarce. Saccharin is usually considered safe and often used as a negative control in toxicological studies, such as the study conducted by Selderslaghs et al. (2009). But the authors actually found that saccharin at 55 mM induced 92 % mortality within 24 h post fertilization in zebrafish embryos. As to ASP, Soffritti et al. (2007) have conducted a study with rats using a dose (100 mg/kg bw/day) higher than the acceptable daily intake value for humans (40 mg/kg bw in the European Union and 50 mg/kg bw in the United States). They found that lifespan exposure to the sweetener is carcinogenic, and exposures beginning from prenatal period further increased the risk. Abd Elfatah et al. (2012) also found that a high dose of ASP at 50 mg daily induced histological lesions and genetic alterations in mother rats and their offspring. But an earlier study found no developmental effect from ASP at much higher doses of 500–4000 mg/kg bw (McAnulty et al. 1989).

Due to the need for more information regarding developmental toxicity and ecological impact of ASWs, we used medaka embryos (*Oryzias latipes*) as a bioassay to tackle both issues at the same time. The fish is an oviparous freshwater teleost. It has become a popular animal model in recent years due to its hardiness, small size (adult 2–4 cm in length), high fecundity (spawn 10–20 eggs daily), and short generational time (2–3 months). Most of all, as medaka embryos are transparent, their development can be easily observed in whole living embryos. These qualities have rendered the fish ideal for developmental toxicity studies.

Here, using medaka embryos, we investigated developmental toxicity of two ASWs, ASP and SAC, along with the natural sweetener sucrose (SUC) and the known developmental toxin-caffeine (CAF). As CAF was often ingested with sweeteners, the combinations of CAF with SUC, ASP, or SAC were tested as well. The concentrations used in this study were much lower than those of the above-mentioned studies because they were intended to be more realistic and environmentally relevant. We also used the white preference test (Lee and Yang 2014) to investigate behavioral consequences of this developmental toxicity. Furthermore, a novel approach—the integrated biomarker response (IBR; Beliaeff and Burgeot 2002; Lee and Lee 2014) was applied to evaluate the overall developmental toxicity of the tested substances.

## Methods

### Experimental animals

A colony of medaka had been established in the current facility for over 5 years. They were maintained at 28 °C under a constant 14 h light:10 h dark photoperiod in glass tanks filled with flow-through filtered water (pH 7.5–7.8). Medaka embryos were incubated in embryo solution (0.1 % NaCl, 0.003 % KCl, 0.004 % CaCl_2_·2H_2_O, and 0.016 % MgSO_4_·7H_2_O, pH 7.5–7.8). All salts in the solution were supplied by J.T.Baker (Phillipsburg, NJ, USA). The fish were fed three times daily with brine shrimp (<24 h after hatching). All procedures were carried out in accordance to the “Guidelines for Animal Experimentation” of Chang Jung Christian University, Taiwan.

### Chemicals and test solutions

Sucrose (PA34230, purity > 99 %) was purchased from Panreac (Barcelona, Spain); ASP (228650050, purity > 98 %), SAC (149001000, purity > 98 %), and CAF (108160100, purity > 98.5 %) were purchased from Acros Organics (Geel, Belgium). A 1000× stock solution of ASP was prepared with DD water. Stock solutions of SAC and CAF were prepared as 10× with embryo solution. All of the stock solutions were then diluted with embryo solution immediately before exposures to make final concentrations of ASP at 0.2 and 1 mM, SAC at 0.005 and 0.05 mM, and CAF at 0.05 and 0.5 mM. The SUC test solutions were prepared at concentrations of 29 and 146 mM. All (stock) solutions were stored at 4 °C and used within a month. The concentrations, abbreviations, and numbers of embryos in each group are listed in Table 1.

**Table 1:** Abbreviations and concentrations of chemicals used in this study

Chemical	CAS number	Log P	LD50 (oral)	Molecular weight	Abbreviation		Concentration		N
					Behavior	Embryonic development	mM	mg/ml	
Sucrose	57-50-1	−3.70^a^	29,700 mg/kg [Rat]^a^	342.3	SUC	SUC1	29	10	15
						SUC2	146	50	14
Aspartame	22839-47-0	−0.10^b^	>10,000 mg/kg [Rat]^c^	294.3	ASP	ASP1	0.2	0.06	15
						ASP2	1.0	0.30	15
Saccharin	81-07-2	0.91^a^	17,000 mg/kg [Mouse]^a^	183.2	SAC	SAC1	0.005	0.001	14
						SAC2	0.050	0.010	14
Caffeine	58-08-2	−0.07^a^	127 mg/kg [Mouse]^a^	194.2	CAF	CAF1	0.05	0.01	14
						CAF2	0.50	0.10	14
–	–	–	–	–	CAF + SUC	CAF2 + SUC1	Refer to individual compounds		11
–	–	–	–	–	CAF + ASP	CAF2 + ASP1			11
–	–	–	–	–	CAF + SAC	CAF2 + SAC1			11
Control	–	–	–	–	–	–	–		38

– not applicable

^a^The Human Metabolome Database, http://www.hmdb.ca/

^b^The DrugBank Database, http://www.drugbank.ca/

^c^TCI America Material Safety Data Sheet, https://www.spectrumchemical.com/MSDS/TCI-A0997.pdf

### Embryo exposures

Medaka embryos from breeding pairs were collected within 5 h post fertilization. They were randomly assigned to different exposure groups, placed in 24-well plates, one embryo per well, and incubated at 27 °C. Each well contained 1 ml embryo or test solutions, which were replaced every 24 h. Embryos treated with embryo solution served as controls. To limit observation period within 2 h, each exposure experiment contained less than 17 embryos (2–4 per group), and the experiment was repeated 13 times. Obviously the 12 treatment groups could not be all tested at the same time, but the control group was always included in each experiment. The embryos were exposed continuously until hatch. The hatchlings were photographed and then transferred to glass dishes containing embryonic solution only.

### Observations and image analysis

The procedures were as described previously (Lee et al. 2012). Briefly, the heart rate of embryos was first counted for 1 min under a dissection microscope at 27 °C, then the embryos were anaesthetized with 0.06 % MS222 (pH 7.25, Sigma-Aldrich), and their images recorded under a microscope (IX2-SLP, Olympus, Tokyo, Japan) from 1 to 3 days post fertilization (dpf) and at hatch. The eye length, width, and pigmentation density (eye density), the distance between the eyes (eye distance; representing the head growth), the width of the optic tectum (midbrain width), and the hatchling body length were analyzed from the images with ImageJ image processing and analysis software (http://rsbweb.nih.gov/ij/). All images were obtained and analyzed under identical conditions without any alteration.

### White preference test

The white preference test was conducted as described previously (Lee and Yang 2014). Briefly, individual hatchling (4 days after hatching) was held with a pipet for 30 s and then released in the black area of a rectangular box (L 60 mm × W 40 mm × H 15 mm) with two equal black and white areas. A square pattern of 5 × 5 mm^2^ was drawn on the bottom surface. Once released, hatchlings were allowed to explore freely for 2 min. Each hatchling was tested once. The following endpoints of the hatchling movement were analyzed from the video recording: time lapse to white area, time spent in black or white areas, number of squares entered (indicating swimming distance), and time swimming along the sides or around the center of the box.

### The integrated biomarker response (IBR)

The integrated biomarker response (IBR) was calculated as described previously (Beliaeff and Burgeot 2002; Lee and Lee 2014). Briefly, measurements from the treatment groups were standardized as a set of indexes, one set for each endpoint. An IBR value was calculated from the indexes of four endpoints (day to hatch, hatchling body length, time lapse to white area in the behavioral test, and total number of squares hatchlings entered in 60 s) from each treatment group.

### Statistical analysis

The statistics software SPSS 17 (SPSS Inc., Chicago, USA) was used for all data analysis. One-way ANOVA was conducted, followed by the LSD post hoc test to compare variables of each endpoint among the treatment groups. Values were considered as significantly different when P <0.05.

## Results

### Effects of exposures on embryonic development

**Fig. 1:**
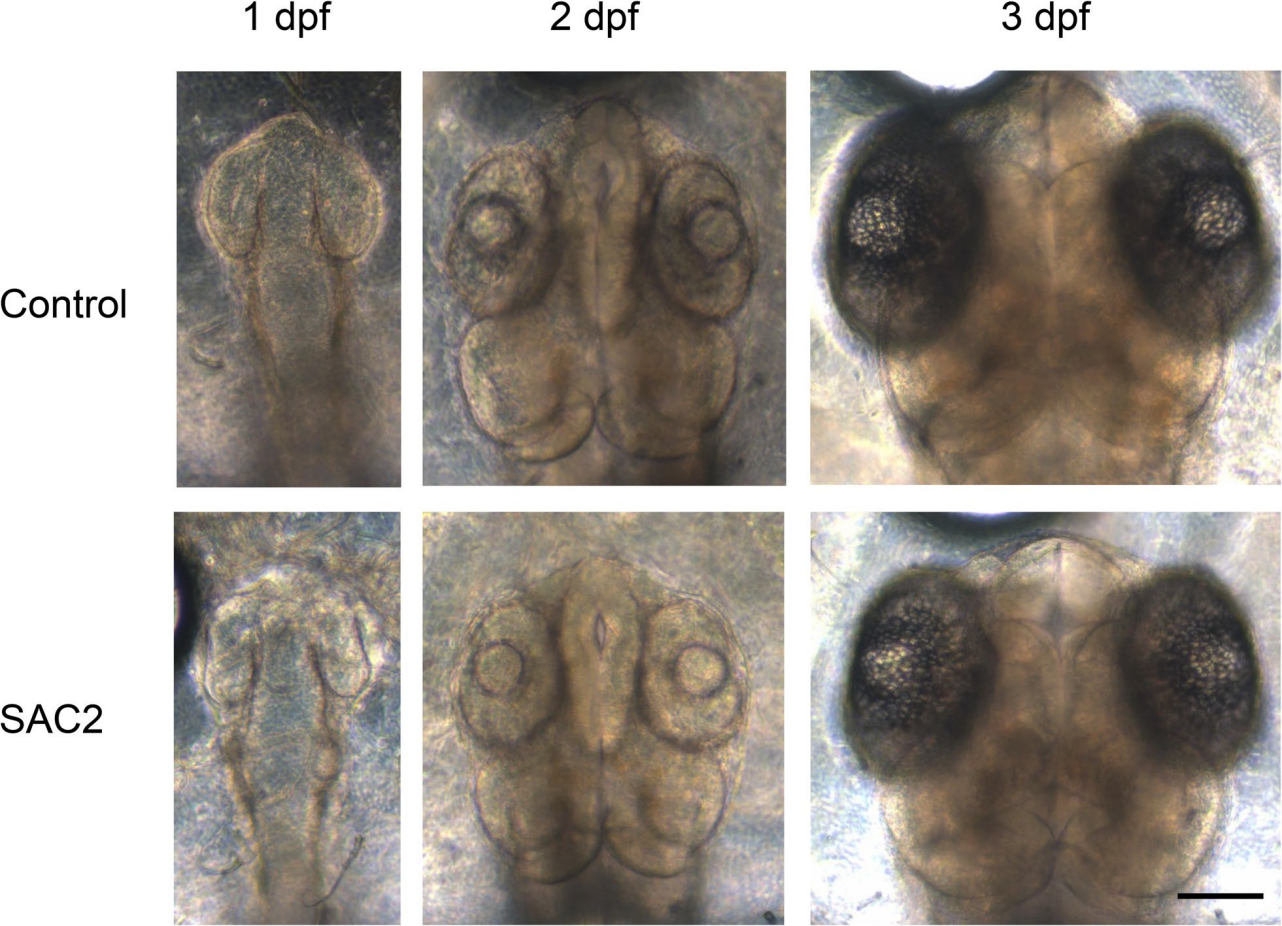
Representative images of medaka embryos exposed to saccharin at 0.05 mM (*SAC2*) or embryo solution only (*control*) at 1–3 days post fertilization (*dpf*). The exposures started from less than 5 h post fertilization until hatch. The images were cropped, but their relative proportion was maintained. No other alteration was made. *Bar* 100 µm

Representative images of the embryos from the control and SAC2 groups are shown in Fig. 1, in which the SAC2-treated embryo appeared to be less developed than the control. The responses of each endpoint are described as follows:

#### Heart rate

**Fig. 2:**
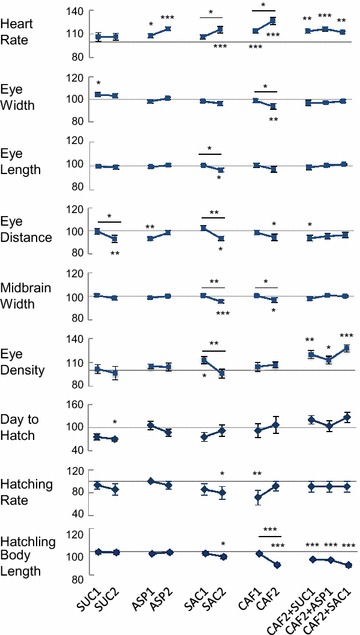
Effects of sucrose (SUC), aspartame (ASP), saccharin (SAC), caffeine (CAF), and CAF combined with each sweetener on medaka development at 3 days post fertilization or at hatch. Data were percents of control values and expressed as mean ± SEM. *Significantly different from the control or between pairs of groups, p < 0.05; **p < 0.01; ***p < 0.001. The abbreviations and concentrations of the substances are listed in Table 1

The heart rate appeared to be the most sensitive endpoint for the tested substances. At 2–3 dpf (Fig. 2; only data at 3 dpf were shown), most of the treatment groups had significantly higher heart rates than the control. The increases of the heart rates ranged widely, from 7.8 % (ASP1) to 26.2 % (CAF2) at 3 dpf. In addition, CAF2 combined with SUC1, ASP1, and SAC1 had significantly lower heart rate than CAF2 alone. In regard to dose-dependence, the pairs of SAC1/2 and CAF1/2 at 3 dpf exhibited such an effect, with higher concentrations causing significantly higher heart rates.

#### Eye width and length

The eye width and length were not affected as much as the heart rate. The eye width of SUC1 increased significantly at 1 and 3 dpf, compared to the control, while that of CAF2 decreased at 2 and 3 dpf (Fig. 2; only data from 3 dpf were shown). However, these differences were small, only up to 6.3 %. A dose dependent effect was found between the pair of CAF1/2 at 3 dpf; CAF2 was 5.1 % smaller than CAF1.

As to the eye length, only ASP1 at 2 dpf (not shown) and SAC2 at 3 dpf (Figs. 1, 2) were significantly shorter than the control. The groups of SAC1/2 also exhibited a dose-dependent effect at 3 dpf: SAC2 was 3.9 % shorter than SAC1.

#### Eye distance

The exposures did not significantly affect the eye distance until 3 dpf, when SUC2, ASP1, SAC2, CAF2, and CAF2 + SUC1 were significantly shorter than the control (Fig. 2). The differences were also small, only up to 6.9 %. A dose-dependent effect was found between the pairs of SUC1/2 and SAC1/2.

#### Midbrain width

At 1 dpf, the midbrain width of CAF1 and CAF2 increased 14.2 and 10.7 %, respectively, compared to the control (not shown). But this increase disappeared at 2 dpf, and at 3 dpf CAF2 became significantly, though only 3.3 %, shorter than the control (Fig. 2). The midbrain width of SAC2 was also significantly shorter than that of the control at 3 dpf. A dose-dependent effect was found between ASP1/2 at 2 dpf (not shown), and SAC1/2 and CAF1/2 at 3 dpf.

#### Eye density

The SAC1 test solution significantly increased (12.9 %) the eye density of the exposed embryos at 3 dpf, compared to the control (Fig. 2). Interestingly, CAF combined with all three sweeteners also raised the eye density significantly; the increases ranged from 12.9 to 27.5 %. A dose-dependent effect was found in SAC1/2; SAC2 averaged 15.2 % lower than SAC1.

#### Day to hatch

Both SUC1 and SUC2 took 23.9 and 30.0 %, respectively, less time to hatch, compared to the control, but this difference was only statistically significant in SUC2 (Fig. 2). Conversely, the CAF2 + SUC1 and CAF2 + SAC1 groups took 20.3 and 26.8 %, respectively, longer time to hatch than the control, but these differences were not statistically significant.

#### Hatching rate

The groups of SAC2 and CAF1 had significantly lower hatching rates than the control (80.0 ± 11.1 and 71.4 ± 13.0 %, respectively, vs. 100 %). The other groups were comparable to the control (Fig. 2).

#### Hatchling body length

The hatchlings from SAC2 and CAF2 were 4.3 and 11.4 %, respectively, shorter than the control. Interestingly, the combinations of CAF2 with the sweeteners caused a 7.2–11.7 % significant reduction in hatchling body length, compared to the control. These hatchlings were also significantly shorter than those exposed to SUC1, ASP1, or SAC1 alone (Fig. 2). A dose dependent effect was seen between the pairs of CAF1/2.

### Effects of exposures on hatchling anxiety-like behavior

**Fig. 3:**
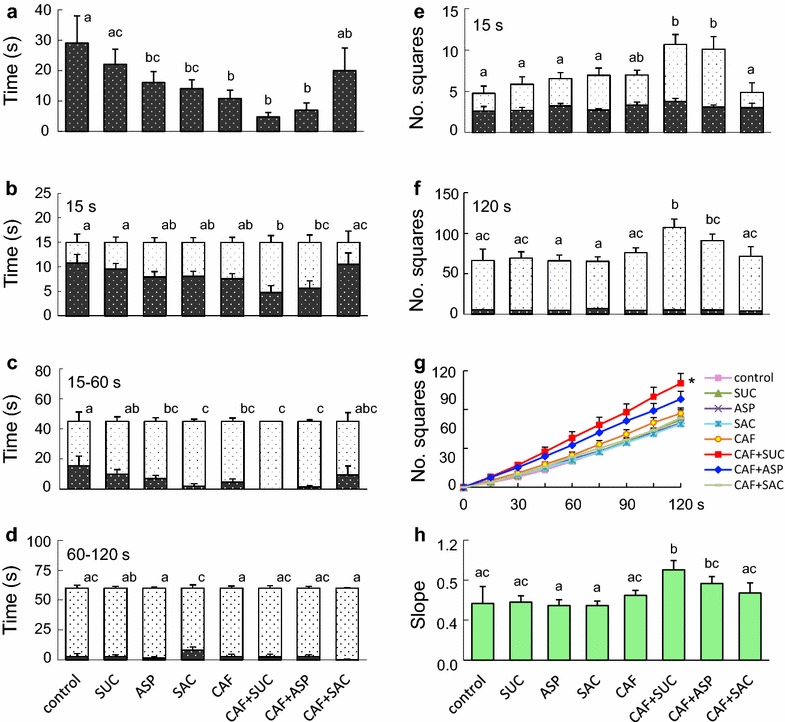
Results of the white-preference test from medaka hatchlings exposed to test substances during embryonic development. **a** Time lapse of the hatchlings crossing from the *black* to *white area*; **b** time distribution of hatchlings in the *black* and *white areas* during the first 15 s, **c** 15–60 s, and **d** 60–120 s; **e** number of squares hatchlings entered during the first 15 s and **f** the whole 120 s; **g** cumulative number of squares hatchlings entered during the 120 s test period; **h** slopes of the curves from (**g**). *Different letters* indicate significant differences, p < 0.05. *Letters on the side of the columns* in **b**–**d** are comparisons of the *white areas*, and those on *top of the columns* in **e** and **f** are comparisons of total number of squares. No letter was placed by the *black areas* in **b**–**d** because statistical analyses produced identical results to those of the *white areas*. In **e** and **f**, no significant difference was found among the *black areas*, while statistical analyses on the white areas produced identical results to those on the total numbers of squares. Values were expressed as mean ± SEM. *Significantly different from the control, p < 0.05. The abbreviations and concentrations of the substances are listed in Table 1

As there was no significant difference between pairs of substances at different concentrations, their data were combined for analyses (Fig. 3). Representative recordings of the white preference test from the control and CAF + SUC hatchlings are available as Additional files 1 and 2.

The result showed that, compared to the control (29.1 ± 8.9 s), most of the treatment groups took significantly less time crossing to the white area (Fig. 3a). The differences of this time lapse ranged from 13.0 s (ASP at 16.1 ± 3.6 s, p < 0.05) to 24.3 s (CAF + SUC at 4.8 ± 1.4 s, p < 0.01). At 15 s (Fig. 3b), CAF + SUC and CAF + ASP spent significantly less time in the black area (4.8 ± 1.4 and 5.6 ± 1.5 s, respectively, vs. 10.8 ± 1.7 s, p < 0.05) and more time in the white area. During 15–60 s (Fig. 3c), in addition to CAF + SUC and CAF + ASP, the groups of ASP, SAC, and CAF were also significantly different from the control (p < 0.05) in their time distribution in the black and white areas. During 60–120 s (Fig. 3d), none of the treated groups were significantly different from the control.

As to swimming distance, at 15 s (Fig. 3e), total numbers of squares from CAF + SUC and CAF + ASP were significantly higher than that of the control (10.7 ± 1.2 and 10.1 ± 1.6, respectively, vs. 4.8 ± 1.3, p < 0.05). At 120 s (Fig. 3f), CAF + SUC remained significantly higher than the control (107.0 ± 10.3 vs. 66.3 ± 13.4, p < 0.01), but not CAF + ASP. Interestingly, the hatchlings exposed to CAF + ASP entered significantly more squares than ASP alone (90.8 ± 8.0 vs. 66.0 ± 7.0, p < 0.05), while those exposed to CAF + SUC also entered significantly more squares than SUC or CAF alone (107 ± 10.3 vs. 69.3 ± 7.5 and 66.0 ± 7.0, p < 0.05, respectively).

The cumulative number of squares hatchlings entered during the test period was shown in Fig. 3g, which indicated that CAF + SUC caused the highest increase among the groups. As shown in Fig. 3h, statistical analyses on the slopes of these cumulative curves produced identical results to those in Fig. 3f.

Interestingly, when in the white area, the hatchlings spent 65.3 ± 2.9 % of the time swimming along the sides, but when in the black area they spent 91.6 ± 1.6 % of the time swimming around the center. This phenomenon was consistent with our previous study (Lee and Yang 2014), but there was no significant difference in this side/center preference among the groups. However, a dose dependent effect was found in the pairs of SAC1/2: when in the white area, the SAC2 group spent significantly more time along the sides of the box, compared to SAC1 (70.3 ± 9.6 vs. 49.0 ± 5.8 %, p < 0.05).

### Integrated biomarker response (IBR) of the treatment groups

To better assess relative developmental toxicity of the substances, the IBRs of the treatment groups were calculated with four endpoints: day to hatch, hatchling body length, time lapse to white area in the behavioral test, and total number of squares hatchlings entered in 60 s.

**Fig. 4:**
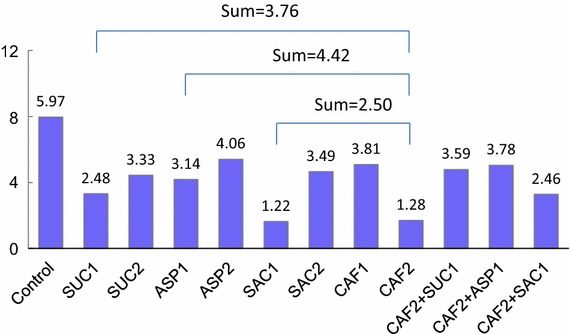
The integrated biomarker response (IBR) values of medaka hatchlings exposed to test substances during embryonic development. The endpoints selected were day to hatch, hatchling body length, time lapse to *white area* in the behavioral test, and total number of squares hatchlings entered in 60 s. The abbreviations and concentrations of the substances are listed in Table 1

As shown in Fig. 4, all of the treatment groups had lower IBRs than the control. The lowest values were from the groups of SAC1 and CAF2, only 20–21 % of the control value. In regard to dose dependence, the group of CAF2 had lower IBR values than CAF1, while SAC2 had higher IBRs than SAC1.

Lastly, to explore the possibility of producing a cumulative effect when CAF was combined with the sweeteners, we compared the actual IBRs of the CAF mixtures with the sums of IBRs from each corresponding individual substance groups. It turned out that the values were quite similar, resulting in a ratio averaged 0.93 ± 0.05.

## Discussion

### Effects of CAF and the sweeteners on embryonic development

The substances of SUC, ASP, SAC, CAF, and the combinations of CAF with the sweeteners all affected embryonic development and/or behavior, as summarized in Table 2.

**Table 2:** Summary of biological responses in medaka embryos exposed to various substances during development

Group	Heart	Eye	Head	Brain	Hatching	Behavior
SUC	√	√	√	–	√	–
ASP	√	–	√	–	–	√
SAC	√	√	√	√	√	√
CAF	√	√	√	√	√	√
CAF + SUC	√	√	√	–	√	√
CAF + ASP	√	√	–	–	√	√
CAF + SAC	√	√	–	–	√	–

– not different from controls

√ significantly different from controls, p < 0.05

As expected, CAF affected development in all six categories listed in Table 2. Surprisingly, all the substances and their combinations affected embryonic heart rates (SUC2 significantly increased the heart rate at 2 dpf, not shown in Fig. 2). In addition, the mixtures of CAF with the sweeteners also advanced the eye development, shortened the hatchling body length, and/or modified behavior.

Caffeine has been known to have developmental toxicity in fish. It had no effect at less than 0.75 mM in zebrafish embryos, but at 1 mM or higher it produced hatchlings with shorter body length (Chen et al. 2008), an effect consistent with our results. Also using zebrafish embryos, Hermsen et al. (2013) reported that CAF at 0.22–1.75 mM induced scoliosis and head and heart malformations. Selderslaghs et al. (2009) also found that CAF at 0.1 mM induced higher incidences of deformed tails, but the hatching rate was only slightly affected. In our study, CAF1 (0.05 mM) caused a 29 % decrease in the hatching rate, but not CAF2 at 0.5 mM. In addition, no apparent malformation was observed from both groups. However, CAF2 significantly shortened the eye and midbrain width and hatchling body length, and raised the heart rates. It is likely that species differences contribute to the inconsistencies, and consequences of CAF biological activities vary with different concentrations and endpoints.

Caffeine is a known psychostimulant; it has been found to activate brain activity and cerebral blood flow in humans, and may induce anxiety in higher doses (Chen and Parrish 2009). Nehlig and Debry (1994) also reported that pregnant rats and mice ingested CAF in doses equivalent to tens of cups of coffee per day produced offspring with altered behavior, including learning abilities and anxiety levels. Recently, Silva et al. (2013) demonstrated that female mice exposed to CAF during pregnancy and lactation produced offspring with increased neuronal network excitability, and the offspring grew up to have cognitive deficits.

However, Brent et al. (2011), after reviewing epidemiological and animal studies, have reported that evidence for developmental toxicity of CAF in humans is inconclusive. The authors indicated that the plasma level of CAF has to reach 0.3 mM to cause teratogenic effects. It is a level roughly equivalent to consuming more than 30 cups of coffee a day in humans, a fairly unlikely scenario. This plasma level of 0.3 mM was in-between the concentrations of CAF1 and CAF2 of this study. Since CAF1 at 0.05 mM already produced effects on medaka development, such as increasing the heart rate and inducing anxiety-like behavior (the time lapses of CAF1 and CAF2 hatchlings moving to the white area were both significantly shorter than that of the control), apparently for subtler effects the minimum plasma level of CAF is likely to be lower.

Other than CAF, SAC was another substance affecting all six categories (Table 2). As SAC also induced significant differences at much lower concentrations than SUC and ASP, it is likely to be more toxic than the other two. This is consistent with the result from a study by Bandyopadhyay et al. (2008), in which SAC at lower concentrations induced more DNA damage than ASP did in mouse bone marrow cells.

Though SAC at 55 mM produced 92 % mortality in zebrafish embryos, it caused no effect at concentrations up to 27.9 mM (Selderslaghs et al. 2009). In another study with zebrafish embryos, SAC at 10 mM did not cause any deformity (Hermsen et al. 2013). In the current study, SAC at a much lower concentration of 0.05 mM caused 20 % mortality, suggesting that, compared to zebrafish, medaka embryos were more sensitive to SAC.

Effects of SUC (or glucose) on development have been studied extensively. It is well-known that diabetic mothers are at five-time higher risk of producing offspring with congenital malformation (Chappell et al. 2009). Glucose exposure at 50 and 100 mM induced malformations and higher than 70 % of mortality in cultured chick embryos (Datar and Bhonde 2005), or impaired neuronal development at 25 mM in quail embryos (Chen et al. 2013). Furthermore, preterm infants given repeated high dose of sucrose were more likely to show poorer attention and motor development (Johnston et al. 2002). In this study, SUC at high concentrations (29 and 146 mM) appeared to have a slight effect on medaka development. But it did not significantly affect hatchlings’ white preference behavior, unless combined with CAF, an issue to be discussed in next section.

### Interaction of CAF and sweeteners in anxiety-like behavior

Our results suggest that SUC is relatively safe at high concentrations. But when combined with CAF, it surprisingly heightened anxiety-like behavior in hatchlings. Furthermore, the mixture of CAF with ASP also significantly raised the anxiety level.

Caffeine has been known to potentiate the reinforcing effects of alcohol through adenosine and dopamine neurotransmission when the two substances are combined (Ferré and O’Brien 2011). Similarly, CAF has been linked to increased additive properties of other abused substances, including cocaine, nicotine, and sugar (Temple 2009). Incidentally, SUC-dependent rats have been shown to have altered dopamine receptors and opioid mRNA levels similar to those in morphine-dependent rats (Spangler et al. 2004). Thus, our result was consistent with the notion that the interaction between CAF and SUC may pose a health risk to younger populations (Seifert et al. 2011). However, whether this interaction represents a synergistic relationship would require further studies using appropriate mixture designs.

The potential toxicity of ASP has been investigated extensively for decades, and so far ASP is still considered very safe (Butchko et al. 2002). More recent studies also reported no evidence supporting a risk to human health, including preterm deliveries (Marinovich et al. 2013), nervous system function, learning, and behavior (Magnuson et al. 2007). But in our study, ASP increased the heart rate in medaka embryos, slightly suppressed the head growth, and induced anxiety-like behavior in hatchlings. Therefore, more studies are needed to ensure the safety of ASP consumption.

### Cumulative effects of CAF and sweeteners

From the IBRs, we found a cumulative effect in developmental toxicity when CAF is combined with the sweeteners. However, further studies are required to confirm the result.

Caffeine is not very soluble in water, due to its non-polar ring structure. Consequently, the molecules tend to self-aggregate and stack with each other on their flat surface like coins (Tavagnacco et al. 2011). In the presence of SUC, CAF would be drawn by a weak affinity to stack with SUC instead, which increases CAF solubility (Lilley et al. 1992). Both ASP and SAC also have ring structures, and they might similarly stack with CAF in solutions. But this is just speculation, and there is no evidence indicating that this structurally stacking contributes to the cumulative effect of CAF with the sweeteners.

The IBR values of the SAC1 and CAF2 groups were the lowest among the groups, while those of ASP and SUC were at the similar level. Since the concentration of SAC1 was much lower than that of CAF2, and that of ASP1/2 was much lower than that of SUC1/2, the ranking of developmental toxicity should be SAC > CAF > ASP > SUC. We have also demonstrated that the IBR is a useful tool to evaluate developmental toxicity with multiple endpoints.

As the concentrations of SAC and ASP tested in this study were at least 10 times higher than those found in the environment, the health risk of these two substances on wildlife may be negligible. However, CAF and other ASWs are increasingly found in aquatic environments. For example, CAF and cyclamate have been found to be at the concentrations of 265–14,418 and 28–1406 ng/L, respectively, in surface water (Tran et al. 2013). Their cumulative activities may amount to significant levels and deserve more investigations.

## Conclusion

We used the medaka embryo as a model system to evaluate developmental toxicity of CAF and three sweeteners: SUC, ASP, and SAC. Several endpoints for development were selected for evaluation, including the heart rate, eye density, time to hatch, and anxiety-like behavior. We found that all four substances and the mixtures of CAF with the sweeteners affected development and/or behavior. We then used the IBR to better evaluate the overall toxicity of these substances. The result showed that the ranking of developmental toxicity was SAC > CAF > ASP > SUC, and there was a cumulative effect when CAF was combined with the sweeteners. Although the concentrations we tested were higher than those detected from the environment, this study has demonstrated that ASWs may pose a health risk to both humans and wildlife, and the effects may accumulate to significant levels when CAF is combined with ASWs.

## Additional files

**Additional file 1.** Representative recording of the white preference test from the hatchling of the control group. The hatchling was treated with embryo solution only during embyronic development.
**Additional file 2.** Representative recording of the white preference test from the hatchling of the CAF + SUC group. The hatchling was treated with embryo solution containing caffeine (CAF) and sucrose (SUC) during embyronic development.
